# Biosynthetic Enhancement of the Detection of Bacteria by the Polymerase Chain Reaction

**DOI:** 10.1371/journal.pone.0086433

**Published:** 2014-01-17

**Authors:** Julie S. Do, Kris M. Weigel, John S. Meschke, Gerard A. Cangelosi

**Affiliations:** 1 AttoDx, Inc., Seattle, Washington, United States of America; 2 Department of Environmental and Occupational Health Sciences, University of Washington, Seattle, Washington, United States of America; University of Illinois at Chicago College of Medicine, United States of America

## Abstract

Molecular viability testing (MVT) was previously reported to specifically detect viable bacterial cells in complex samples. In MVT, brief nutritional stimulation induces viable cells, but not non-viable cells, to produce abundant amounts of species-specific ribosomal RNA precursors (pre-rRNA). Quantitative polymerase chain reaction (qPCR) is used to quantify specific pre-rRNAs in a stimulated aliquot relative to a non-stimulated control. In addition to excluding background signal from non-viable cells and from free DNA, we report here that MVT increases the analytical sensitivity of qPCR when detecting viable cells. Side-by-side limit-of-detection comparisons showed that MVT is 5-fold to >10-fold more sensitive than standard (static) DNA-targeted qPCR when detecting diverse bacterial pathogens (*Aeromonas hydrophila, Acinetobacter baumannii, Listeria monocytogenes, Mycobacterium avium,* and *Staphylococcus aureus*) in serum, milk, and tap water. Sensitivity enhancement may come from the elevated copy number of pre-rRNA relative to genomic DNA, and also from the ratiometric measurement which reduces ambiguity associated with weak or borderline signals. We also report that MVT eliminates false positive signals from bacteria that have been inactivated by moderately elevated temperatures (pasteurization), a condition that can confound widely-used cellular integrity tests that utilize membrane-impermeant compounds such as propidium iodide (PI) or propidium monoazide (PMA) to differentiate viable from inactivated bacteria. MVT enables the sensitive and specific detection of very small numbers of viable bacteria in complex matrices.

## Introduction

The polymerase chain reaction (PCR) is an integral tool in research, clinical, and environmental microbiology. Although the method has become significantly more reliable and user friendly in recent years, its sensitivity remains problematic when targeted to nucleic acids that are present in very small numbers. Background signal levels can be substantial due to the presence of dead cells or free DNA. Positive results are muted by inhibitory substances in complex biological and environmental samples. Signals that hover near the background level are common in molecular testing, especially in microbiology. In many cases weak signals must be considered negative or indeterminate. For many applications, PCR remains insufficiently sensitive for stand-alone use in microbial pathogen detection. Examples include the diagnosis of sterile-site infections such as bacterial meningitis, smear-negative tuberculosis, and many tasks in biodefense and food/water safety.

In order to improve the specificity of PCR for viable microbial cells, we developed assays for bacterial rRNA precursors (pre-rRNA) [1], [2]. Pre-rRNAs are intermediates in rRNA synthesis with leader and tail fragments that are enzymatically removed to yield mature rRNA. In growing bacteria, pre-rRNAs can account for 25% of total cellular rRNA [3]. They are considerably easier to detect than even the most strongly-expressed mRNAs. When growth slows, pre-rRNA synthesis declines but maturation continues, resulting in active drainage of pre-rRNA pools [4]. Pre-rRNA is rapidly replenished when growth-limited cells are given fresh nutrients [4]–[6]. These changes occur consistently in viable bacterial cells but are not seen in dead cells or with free nucleic acids. Growth-related changes in mature rRNA copy number are far exceeded by those of pre-rRNA [3], [4], [7], [8]. Pre-rRNA sequences are hypervariable and highly species-specific, such that pre-rRNA-targeted PCRs can detect and assess the physiology of individual species in complex samples [2], [3], [7], [9].

All or nearly all bacteria synthesize pre-rRNA upon nutritional stimulation. We have exploited this fact in a method termed molecular viability testing (MVT) or ratiometric pre-rRNA analysis [1], [2]. In this method, a sample is split into two equal aliquots, one of which is nutritionally stimulated, usually by the addition of broad-spectrum bacteriological culture media. If viable cells of a targeted species are present in the sample, then pre-rRNA copy number (measured by reverse transcriptase-quantitative PCR; RT-qPCR) is seen to rapidly increase in the stimulated aliquot relative to the control (non-stimulated) aliquot. Because non-viable cells cannot catalyze this increase, the method selectively detects viable bacteria [1], [2]. Pre-rRNA stimulation is very rapid. One to two hours of nutritional stimulation is adequate for consistent pre-rRNA upshift in most organisms. Slow-growing species such as some *Mycobacterium* spp. (generation time >20 hours) need 4 to 6 hours of stimulation (for convenience of work flow, these samples were usually incubated overnight). In all cases these time periods are ∼1 generation time or less. Thus, MVT is not bacteriological culture, and it does not define bacterial viability by the ability to divide and form progeny. Instead, it defines viability as the ability to synthesize a macromolecule in response to nutritional stimulation.

Previous reports demonstrated that MVT is effective at differentiating viable cells of diverse bacterial species from cells that were inactivated by chlorine [2] or serum exposure [1]. The current report evaluates a different parameter, namely the analytical sensitivity (limit of detection) of MVT relative to standard (static) DNA detection. A previous study [1] suggested that MVT is sensitive (limit of detection ≤56 cfu/mL of *A. baumannii* in human serum). The current study asked whether this observation is generalizable to physiologically and phylogenetically diverse bacteria present in diverse sample types. These measurements were made in side-by-side limit-of-detection comparisons with static DNA-targeted PCR.

The current report also demonstrates that MVT reliably distinguishes viable from inactivated bacteria in pasteurized milk products. In low-temperature pasteurizations, materials are exposed to moderately elevated temperatures for defined time periods (typically 63°C for 30–60 min). These conditions are important to food safety and can serve as models for thermal inactivation in nature. In many bacteria, moderately elevated temperatures can irreversibly denature critical enzymes or organelles, thereby inactivating the bacteria, while leaving the cell envelope intact. Such inactivation mechanisms can confound widely-used molecular viability tests that correlate viability with cell envelope impermeability [10], [11]. Examples include LIVE/DEAD® cell viability assays [Life Technologies, Inc.] and viability PCR, both of which use membrane-impermeant propidium dyes as “probes” to label and/or inactivate DNA in inactivated cells [12]–[14]. The current report tested the hypothesis that MVT, which measures biosynthetic responses to an environmental stimulus rather than cell envelope impermeability, is useful for rapidly assessing bacterial inactivation under such conditions.

## Materials and Methods

### Bacteriological culture

Bacterial strains and conditions for routine culture (as opposed to nutritional stimulation) are listed in Table 1. *M. avium* cultures reached a stationary phase concentration of 10^8^ – 10^9^ cfu/mL after growth in M7H9 for 7 days. All other organisms reached a stationary phase concentration of approximately 10^9^ cfu/mL after incubation in appropriate media for 18–24 hours. Cell densities were determined through viable cell plating.

**Table 1:** Bacterial strains, routine culture conditions, primers, and probes for qPCR and RT-qPCR.

Bacterial species	strain	Culture medium^1^	Fwd-Rev Primers^2^	Probe [6∼FAM]-[TAMRA∼6∼FAM]^2^
*Acinetobacter baumannii*	ATCC 17978	TSB	F2:TGATTGATTGGTTTAAATTACTCGAAG R1:CGCTCGACTTGCATGTGTTA	P2:TGAGCCAGAATTGGCACCTTGTCT
*Aeromonas hydrophila*	ATCC 7966	TSB	F:ATTTGAATCAAGCAATCTGTG R:GTTCAATCTGAGCCATGATC	P1:TGGGCACTCACAGCATCGAGCATC
*Listeria monocytogenes*	BCSI 1691	BHI	F:GGTGAAGTCGTAACAAGG R:CACAGGTTTCCTTTTCCTTAG	P1:TGATCCAGCCGCACCTTCCG
*Mycobacterium avium*	104	M7H9	F:CTCAATAGTGTGTTTGGTCT R:GACTTGCATGTGTTAAGCAC	P1:TGGCCATACCTAGCACTCCCCGTG
*Salmonella enterica*	ATCC 13076	NB	F1:GACAATCTGTGTGGGCAC R4:TCGACTTGCATGTGTTAGG	P1:TGGCTCAGATTGAACGCTGGCGG
*Staphylococcus aureus – RNA amplification*	ATCC 29213	TSB	F2:AACTGAATACAATATGTCACG R2:CGAAGGTGGGACAAATGATT	P2:CCGCATCTTCTGAAGAAGATGTTCCGA
*Staphylococcus aureus - DNA amplification*	ATCC 29213	TSB	2F:CGCATCTTCTGAAGAAGATGTTCCG 3R:GACAAATGATTGGGGTGAAGTCGTA	P1:AGCCGCACCTTCCGATACGGCTACC

^1^ TSB, trypticase soy broth; BHI, brain-heart infusion broth; M7H9, Middlebrook 7H9 broth with OADC enrichment; NB, nutrient broth. All cultures were grown under air at 37°C except *A. hydrophila,* which was grown at 28°C.

^2^ Pre-rRNA and DNA amplifications used the same primer sets for all organisms except *S. aureus,* which required the design of an alternative ribosomal DNA-targeted primer set for optimal DNA detection by qPCR.

### Limit of detection analyses

For analysis of bacteria in milk, cultured bacteria were spiked by serial dilution into a commercial dairy milk product (Horizon 1% Organic ultra-high temperature [UHT] processed milk). In each experiment, five different cell densities plus a control sample with no spiked cells were assessed by MVT and qPCR. Initial cell densities were estimated based on culture densities, then measured more accurately upon completion of each experiment by viable plate counting (colony forming units, cfu) of the most concentrated suspension. Serial dilutions in milk were incubated overnight at 4°C, a typical storage temperature for commercial dairy milk. One mL samples were then taken from each dilution, pelleted by centrifugation, and washed two times in 1 mL PBS. After the final wash, pellets were resuspended in 1 mL PBS, then 100 µL aliquots were transferred in triplicate to 900 µL of PBS (unstimulated) in 1.5 mL tubes, and to 900 µL of culture media (stimulated) in 14 mL tubes. Aliquots transferred to PBS were immediately pelleted, with supernatant removed, and frozen at –80°C for subsequent MVT and qPCR analysis. Stimulated aliquots were briefly incubated with aeration as described in Results. The stimulated aliquots were transferred to 1.5 mL tubes then pelleted, supernatants were removed, and pellets were frozen at –80°C for subsequent MVT analysis. During the stimulation period, samples of the washed and resuspended cell suspension from the most concentrated (least dilute) sample were serially diluted and plated onto appropriate agar media for viable cell counts (colony forming units [cfu]/mL).

For analysis of *A. hydrophila* cells in water, 50 µL samples of stationary phase cultures (approximately 1E9 cfu/mL) were transferred to 3 mL of autoclaved tap water in 14 mL tubes. The samples were incubated for 7 days at 28°C with aeration as described previously [2], then serially diluted in autoclaved tap water for MVT, qPCR, and viable plating analysis as described above for milk samples. For analysis of *A. baumannii* in human serum, 50 µL samples of stationary phase cultures (approximately 1E9 cfu/mL) were spiked into 2 mL serum (Lonza BioWhittaker Normal Human Serum Fisher Scientific BW14-402E) in 14 mL tubers. Samples were incubated with aeration for 2 days at 37°C, then serially diluted in fresh serum for MVT, qPCR, and viable plating analysis as described for milk samples.

### Selective detection of viable bacteria in milk

Stationary phase cultures grown as described above were diluted into 20 mL of Horizon 1% Organic UHT processed milk to approximate final densities ranging from 10^3^ to 10^6^ cfu/mL. Spiked milk suspensions were incubated at 4°C and samples were taken at days 0, 1, and 2 after the addition of bacteria. After taking the day 2 sample, four 1-mL samples of the spiked milk suspension was pasteurized in 1.5 mL tubes at 63°C for 45 minutes in a temperature block, then cooled on ice, combined into a 14 mL tube, and stored at 4°C. Samples were taken from the pasteurized milk suspension directly after pasteurization and cooling (day 2+P), and an additional timepoint was taken one day later (day 3+P). At each sampling, 1 mL of suspension was transferred to a 1.5 mL tube and was pelleted and washed two times with 1 mL of PBS. After the final centrifugation, pellets were resuspended in 1 mL of PBS. Portions of these suspensions were diluted and plated onto appropriate media plates for viable cell counts. In addition, 100 µL aliquots of the resuspended cells were transferred in triplicate to 900 µL of PBS for non-stimulated aliquots and 900 µL culture media for stimulated aliquots (TSB for *S. aureus* and *S. enterica,* and Middlebrook 7H9 for *M. avium*). Stimulated aliquots were incubated with aeration for 90 min (*S. aureus* and *S. enterica*) or overnight (*M. avium*). Stimulated and unstimulated samples were pelleted, supernatant removed and stored at –80°C prior to analysis.

### Nucleic acid extraction using the Qiagen AllPrep Kit

In most experiments total nucleic acid (DNA and RNA) was extracted from pellets stored at –80°C using Qiagen AllPrep Total Nucleic Acid kits. Protocols were modified prior to loading samples onto spin columns, as follows. For *L. monocytogenes* and *M. avium*, 100 µL of TE and 5 µL of lysozyme (50 µg/mL; Thermo Scientific) were added to each pellet. For *S. aureus*, an additional 5 µL of 5 µg/µL lysostaphin (Sigma L7386) was added to each pellet. Samples were vortexed for 30 seconds, then incubated at 37°C for 30 minutes. Subsequently, 350 µL (Gram-negative), 250 µL (Gram-positive) or 280 µL (*M. avium*) of TE + 1% SDS, and 10 µL (20 mg/mL) proteinase K was added to each sample. Suspensions were vortexed and incubated at 60°C for 10 minutes, then 350 µL of Qiagen Buffer RLT Plus was added to each sample. For all bacteria with the exception of *M. avium*, the entire volume of sample was transferred to a Qiagen DNA spin column and the remaining steps of the Qiagen AllPrep protocol were followed. For *M. avium*, the entire sample volume was transferred to a 2 mL screw-cap tube with 50 mg of acid washed beads (Sigma G1145). Samples were bead-beat 3 times at 5 minutes each using a vortex adapter (MO BIO 13000-V1-24), with a 1 minute incubation on ice between treatments. After bead-beating, samples were centrifuged at 13,000 rpm for 10 seconds. Subsequently, 650 µL of the supernatant was transferred to a Qiagen DNA spin column and the remainder of the Qiagen AllPrep protocol was followed. Columns for both RNA & DNA were eluted with 100 µL of TE for samples, with the exception of *M. avium*, which were eluted in 50 µL TE for limit of detection experiments.

### Nucleic acid extraction using Nucleic Acid Extractions Cards

In some experiments, total nucleic acid was extracted from frozen bacterial cell pellets by using the BCSI Nucleic Acid Extraction System [15]. Pellets were briefly thawed at room temperature, and 380 µL of TE + 1% SDS (lysis buffer) and 10 µL of 20 mg/mL Proteinase K (VWR IB05406) were added to each pellet. The suspension was vortexed for 20 seconds, and placed in a 60°C incubator for 10 minutes. Subsequently, 400 µL each of GT Lysis Buffer and 100% ethanol were added to the tube with brief vortexing after the addition of each reagent. The entire volume was loaded onto a nucleic acid extraction (NAE) card and incubated at room temperature for 15 minutes. The cards were loaded onto the NAE automated system with the following wash parameters: 3×1 mL Wash 1; 3×1 mL Wash 2; and 4 minutes of drying time. Cards were manually eluted with 100 µL elution buffer (TE).

### Oligonucleotide Primer and Probe Design

Pre-rRNA and DNA amplifications used the same primer sets for all organisms except *S. aureus,* which required the design of an alternative ribosomal DNA-targeted primer set for optimal DNA detection by qPCR. For all organisms, RT-qPCR primer sets were designed to straddle the 5’ mature rRNA terminus as described previously [1], [2]. Primers for cDNA synthesis and reverse qPCR primers recognized sequences within the mature rRNA (16S), while forward primers recognized species-specific sequences within the 5’ leader region of the pre-rRNA. Therefore, amplification required intact pre-rRNA molecules as templates. In the present study, probe-based qPCR was used as described [1]. Probes recognized sequences within the 5’ pre-rRNA leader region except in *L. monocytogenes* and *S. enterica,* which had relatively confined sequence-specific pre-rRNA regions and required the use of probes that hybridize to mature rRNA. Primer and probe sequences are listed in Table 2. Genome sequences were obtained from NCBI, and all primers and probes were ordered from Eurofins MWG Operon.

**Table 2:** Lower limits of detection of MVT and static qPCR.

Organism	Sample matrix	Nucleic acid extraction method^1^	Range of cell densities tested (cfu/mL)	Nutritional stimulation Conditions^2^	MVT (pre-rRNA) LOD^3^	qPCR (DNA) LOD^4^
*Acinetobacter baumannii*	Serum	NAE	26-2600	90 min in TSB	260	2600
*Acinetobacter baumannii*	Serum	Qiagen	14-1400	90 min in TSB	70	70
*Aeromonas hydrophila*	Tap water	NAE	268-26800	90 min in TSB	2680	26800
*Aeromonas hydrophila*	Tap water	NAE	55-5500	90 min in TSB	110	550
*Listeria monocytogenes*	Milk	Qiagen	10-1000	90 min in TSB	10	100
*Listeria monocytogenes*	Milk	Qiagen	16-3250	90 min in TSB	162	162
*Mycobacterium avium*	Milk	Qiagen	76-7600	Overnight in 7H9	76	3800
*Mycobacterium avium* 5	Milk	Qiagen	50-5000	Overnight in 7H9	100	500
*Staphylococcus aureus*	Milk	Qiagen	26-2640	90 min in TSB	26	264
*Staphylococcus aureus*	Milk	Qiagen	175-17500	90 min in TSB	175	175

^1^ NAE, flat-glass Nucleic Acid Extraction cards (BCSI); Qiagen, Qiagen AllPrep Total Nucleic Acid kit.

^2^ TSB, trypticase soy broth; 7H9, Middlebrook 7H9 broth with ADC enrichment. All stimulations at 37°C except *A. hydrophila,* which was stimulated at 30°C.

^3^ LOD, limit of detection. The LOD of MVT was the lowest density of spiked bacteria to exhibit a positive result, defined as ΔCt > 1 in three out of three replicates. MVT was always negative in the absence of spiked bacteria.

^4^ LOD, limit of detection. The LOD of qPCR was the lowest density of spiked bacteria to exhibit a positive result, defined as a stronger signal (lower Ct value) than all three no-bacteria controls, in three out of three replicates.

^5^ Experiment depicted graphically in Figure 1.

### qPCR and RT-qPCR

qPCR was performed on an Applied Biosystems 7500 Fast Real-Time PCR System using the Thermo Verso 1-step QRT-PCR kit (AB-4100/C) for RNA measurement and Roche FastStart Universal Probe Master (ROX) (Part No. 04913949001) for DNA measurement. Cycling parameters for RNA PCR were as follows: 50°C for 30 minutes, 95°C for 15 minutes, and 40 cycles of 95°C for 15 seconds and 60°C for 1 minute. Cycling parameters for DNA PCR were as follows: 50°C for 2 minutes, 95°C for 15 minutes, and 40 cycles of 95°C for 15 seconds and 60°C for 1 minute. For both DNA and RNA PCR, suggested volumes were used as described in product guides. A final concentration of 400 nM was used for primers, 200 nM for probe, and 200 nM for the ROX passive reference dye (RT-qPCR), with 5 µL of each extraction template added.

## Results

### MVT enhances the sensitivity of detection of bacteria in complex matrices

To evaluate the sensitivity of MVT relative to static (standard) DNA-targeted qPCR, both assays were applied to cells of diverse bacterial species, namely *Aeromonas hydrophila, Acinetobacter baumannii, Listeria monocytogenes, Mycobacterium avium,* and *Staphylococcus aureus*. To simulate relevant contaminated samples, bacteria were spiked at varying concentrations into tap water, human serum, or dairy milk prior to analysis.

In contrast to qPCR, which measures the static copy number of target DNA molecules, MVT measures a change in bacterial physiology, namely increased pre-rRNA copy number in response to nutritional stimulation. The measurement is made by comparing Ct values generated by RT-qPCR analysis of samples before and after nutritional stimulation. As in previous studies [1], [2], RT-qPCR primers straddled the junction between the 5’ terminus of the mature rRNA and the pre-rRNA leader region, such that intact pre-rRNA molecules are required as templates. Upon nutritional stimulation, a reduction in pre-rRNA Ct value of ≥1.0 relative to the non-stimulated control (ΔCt ≥ 1.0) indicates that pre-rRNA was synthesized. This constitutes a positive MVT result, irrespective of the absolute quantities of target pre-rRNA present. In the present study each MVT measurement was run in triplicate and samples were considered positive by MVT when all three replicates exhibited ΔCt ≥ 1.0.

To compare the sensitivity of MVT to static DNA-targeted qPCR, total nucleic acid (TNA) was extracted from spiked samples by using protocols designed to extract both DNA and RNA from bacteria with similar efficiencies. Two different commercial TNA extraction methods were used in various experiments, thereby minimizing artifacts that might result from uneven DNA and RNA extraction. Static qPCR was conducted in triplicate on non-stimulated aliquots, consistent with standard PCR detection strategies. Milk and tap water samples occasionally had naturally-occurring nucleic acid of targeted species, resulting in positive qPCR results in the absence of spiked bacteria (MVT results were always negative in the absence of spiked bacteria). A spiked sample was considered positive by qPCR when all three replicates delivered stronger qPCR signals (lower Ct) than all three non-spiked control samples. Simultaneously with MVT and qPCR measurements, samples were plated onto bacterial culture medium to measure actual viable counts of the targeted bacteria.

**Figure 1:**
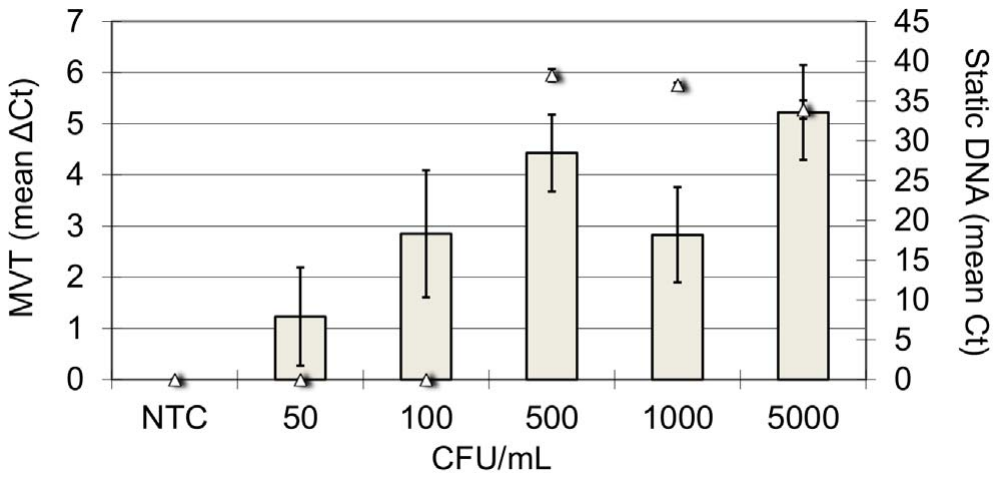
Detailed results of a typical experiment. The results correspond to the second *M. avium* experiment shown in Table 2. Grey bars are MVT results (mean ΔCt ±SD from the triplicate measurement). Triangles are static DNA results (mean Ct ±SD from the triplicate measurement). NTC, milk with no spiked bacteria.

The results are summarized in Table 2, and Figure 1 shows a typical experiment in greater detail. In 7 out of 10 experiments, MVT was 5-fold to >10-fold more sensitive than static qPCR. The two methods exhibited similar sensitivity in 3 of the 10 experiments. In no experiments was MVT less sensitive than static qPCR. In some samples, such as the first of two tap water samples spiked with *A. hydrophila* (Table 2), there were signs of PCR inhibition, which decreased the analytical sensitivities of both MVT and static qPCR. However, the former method was still more sensitive than the latter. The increased sensitivity of MVT was evident across all bacterial species, all sample matrices, and both TNA extraction methods used in these experiments.

### Distinguishing viable from inactivated cells after low-temperature pasteurization

We previously reported that MVT distinguished viable from chlorine-killed cells of *A. hydrophila* in tap water [2], and viable from serum-killed cells of *Pseudomonas aeruginosa* in human serum [1]. The current project asked whether MVT can distinguish viable bacterial cells from cells that have been inactivated by low-temperature pasteurization (63°C for 45 minutes), a condition that is known to leave cell envelopes intact as indicated by membrane-impermeant dyes [10], [11].

**Figure 2:**
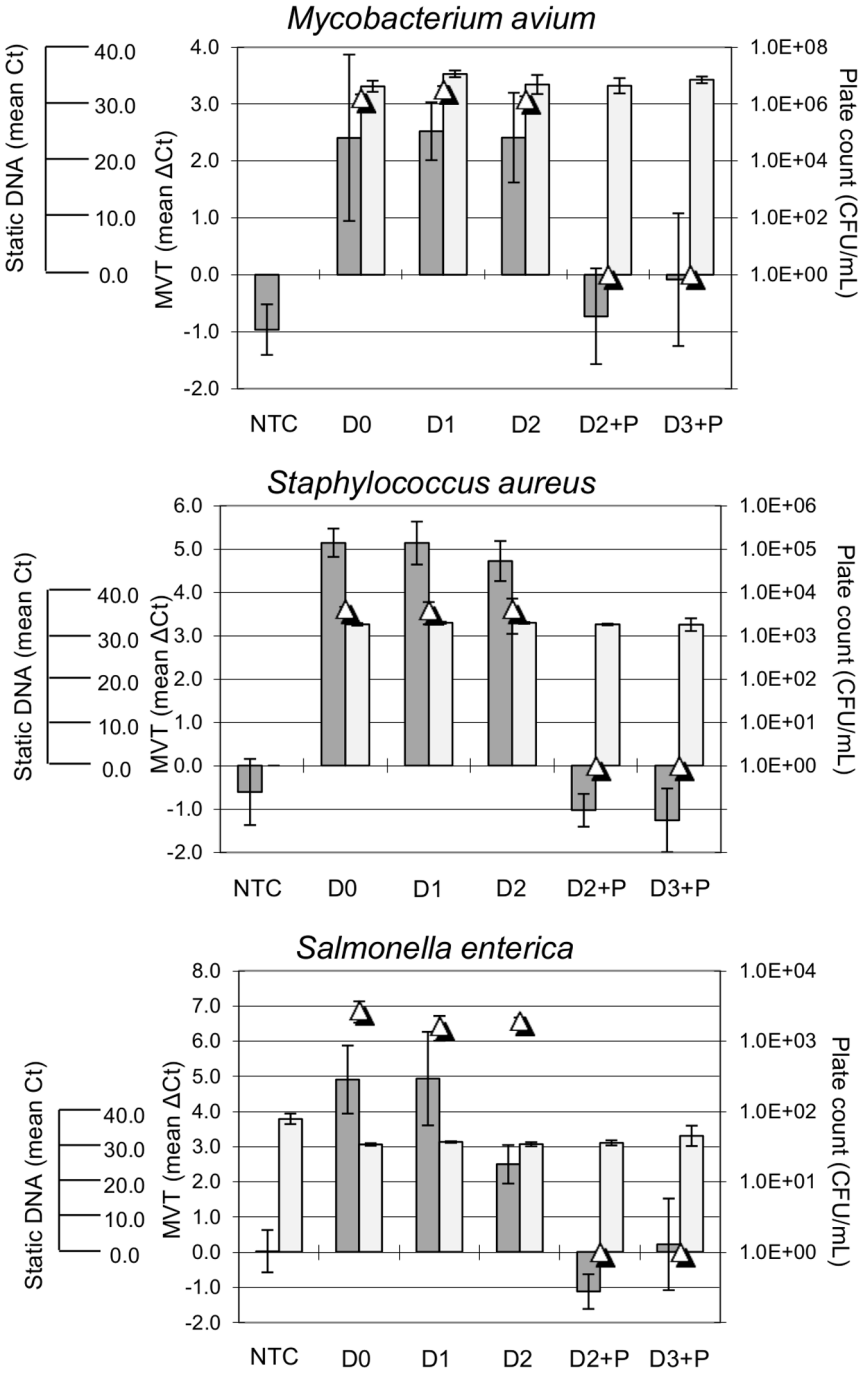
Molecular viability testing in milk. Spiked milk suspensions were incubated at 4°C and samples were taken on days 0, 1, and 2 after addition of bacteria (D0, D1, and D2). After the day 2 sample, the spiked suspensions were pasteurized at 63°C for 45 minutes then cooled on ice. Samples were taken from the pasteurized milk suspensions immediately after cooling (D2+P) and one day later (D3+P). Dark bars, MVT results. Light bars, static DNA results. Triangles, viable plate counts. NTC, milk with no spiked bacteria.

A relevant biological matrix, commercial ultra-high temperature (UHT) pasteurized dairy milk, was spiked with cultured cells of *M. avium, S. aureus, and Salmonella enterica.* Suspensions were incubated at 4°C and pre-inactivation samples were taken at days 0, 1 and 2. Bacteria in samples were pelleted and washed in PBS to remove PCR inhibitors. Washed samples were subjected to MVT analysis and to static qPCR as described in Methods. Samples were also plated to confirm the presence or absence of viable cells. All three organisms showed clear positive MVT results (ΔCt ≥ 1.0) on Days 0, 1, and 2, when viable cells were present (Figure 2).

After Day 2 sampling, the suspensions were subjected to low-temperature pasteurization. Samples were taken immediately after pasteurization (Day 2+P) and on the following day (Day 3+P). Pasteurized samples yielded no growth on culture plates and consistently negative results by MVT. In contrast, static qPCR was positive before and after pasteurization, consistent with persistence of pathogen DNA either extracellularly or within inactivated cells (Figure 2). In addition, the *S. enterica* qPCR yielded a false-positive DNA signal from un-spiked milk (NTC in Figure 2). Background DNA may have been present in the sample, given the common occurrence of *Salmonella* in bulk tank milk [16]. Alternatively, it may have been present in the PCR reagents (the targeted pre-rRNA sequences are identical in *Escherichia coli,* which is used in the production of recombinant Taq polymerase used in PCR). Regardless of the source of this false-positive result, the MVT method effectively eliminated it.

## Discussion

Under ideal conditions PCR can detect a single nucleic acid molecule. However, when applied to complex natural samples, the sensitivity of PCR is blunted by inhibitors, non-target nucleic acids, and nucleic acids associated with inactivated or lysed target cells. For example, extensively optimized commercial qPCR tests for *Mycobacterium tuberculosis,* methicillin-resistant *S. aureus* (MRSA), and *Clostridium difficile* in patient samples have reported limits of detection (LOD) ranging from 130 cfu/mL to over 2,000 cfu/mL, despite sample processes that volumetrically concentrate pathogen cells for detection [17]–[19]. These analytical sensitivities fall well short of the theoretical LODs of qPCR under ideal conditions. For many applications in biodefense, food and water safety, and clinical diagnosis (e.g sterile site infections such as meningitis, and smear-negative tuberculosis), PCR is not considered sensitive enough to serve as a stand-alone pathogen detection tool. MVT was developed in part to address this shortfall. In diverse bacterial species including gram positive species, gram negatives species, and slow growing mycobacteria, MVT was 5- to 10-fold more sensitive than static DNA-targeted qPCR in most experiments.

Pre-rRNA is significantly more abundant than genomic DNA in stimulated bacteria. In one study, pre-rRNA was estimated to account for 25% of total rRNA in growing *Acinetobacter* cells [3], consistent with thousands of copies per genome. Thus, a single stimulated bacterial cell can release numerous copies of pre-rRNA for analysis, an advantage when analyzing samples with very small cell counts. Despite this advantage, in many experiments described here and previously [1], [2] RT-qPCR signals from pre-rRNA were not significantly stronger than qPCR signals from genomic DNA amplification. This can be explained by the lability of RNA relative to DNA, and by the inefficiency of cDNA generation by reverse transcriptase. However, even when RT-qPCR signals are weak, MVT is consistently sensitive. In contrast to standard qPCR, MVT measures a change in bacterial physiology, namely the dramatic increase in pre-rRNA copy number that occurs upon nutritional stimulation of viable bacterial cells. This “dynamic sensitivity” is analogous to the observation of animals in a forest, in that moving animals are easier to see than stationary ones. Pre-rRNA synthesis is a type of bacterial “movement” that can be reliably induced by nutritional stimulation, resulting in improved resolution of borderline samples, which are frequently encountered in diagnostics and other microbiological detection tasks.

In addition to demonstrating biosynthetic enhancement of PCR sensitivity, the present study extended previous findings [1] that demonstrated MVT detection of viable cells in complex biological matrices (serum in the previous report, milk in the current report). Such environments have nutrients which could, in theory, enable bacterial replication and the maintenance of large pre-rRNA pools. This could diminish the resolving power of MVT, which detects the upshift of pre-rRNA synthesis upon nutritional stimulation. However, in nature microbial growth is nearly always limited by the availability of at least one nutrient. Nutritional stimulation conditions that provide limiting nutrient(s) may stimulate pre-rRNA synthesis in bacteria derived from most, if not all, natural environments.

As a tool for distinguishing viable from inactivated microorganisms in samples, an effective and widely used alternative to MVT involves incubating bacteria with membrane-impermeant DNA stains such as propidium monoazide (PMA) or propidium iodide (PI). Exclusion of these reagents from viable cells is detectable by “viability PCR”, in which PMA renders DNA in compromised cells inactive to PCR amplification [12], [13], or by fluorescence microscopy or cell sorting, in which DNA staining in compromised cells is detected fluorometrically [14]. In both methods, viability is correlated with cell envelope impermeability. A limitation is that some inactivated cells retain intact cell envelopes that exclude the reagents, resulting in false-positive “viable” results under some conditions [20], [21]. This has been reported to occur in low-temperature pasteurization [10], [11], a condition that is important to food safety and a model for naturally-occurring bacterial inactivation by extended exposure to moderately elevated temperatures. In the current study, MVT consistently detected bacterial inactivation by low-temperature pasteurization. This was observed in a gram positive species, a gram negative species, and a slow growing *Mycobacterium* species, suggesting that the observation may be generalizable..MVT may be effective under these conditions because it defines viability as the ability to synthesize a macromolecule in response to a stimulus, a relatively early-stage manifestation of bacterial inactivation [14], [22].

In summary, we report that MVT significantly increases the resolving power, and thereby the analytical sensitivity and specificity, of PCR detection of diverse bacterial pathogens in human and environmental sample types. We also report that MVT detects bacterial inactivation by low-temperature pasteurization. The method may prove to be a useful addition to the molecular microbiological toolbox.
